# Why vision is not both hierarchical *and* feedforward

**DOI:** 10.3389/fncom.2014.00135

**Published:** 2014-10-22

**Authors:** Michael H. Herzog, Aaron M. Clarke

**Affiliations:** Laboratory of Psychophysics, Brain, Mind Institute, École Polytechnique Fédérale de LausanneLausanne, Switzerland

**Keywords:** feedback, object recognition, crowding, Verniers, Gestalt

## Abstract

In classical models of object recognition, first, basic features (e.g., edges and lines) are analyzed by independent filters that mimic the receptive field profiles of V1 neurons. In a feedforward fashion, the outputs of these filters are fed to filters at the next processing stage, pooling information across several filters from the previous level, and so forth at subsequent processing stages. Low-level processing determines high-level processing. Information lost on lower stages is irretrievably lost. Models of this type have proven to be very successful in many fields of vision, but have failed to explain object recognition in general. Here, we present experiments that, first, show that, similar to demonstrations from the Gestaltists, figural aspects determine low-level processing (as much as the other way around). Second, performance on a single element depends on all the other elements in the visual scene. Small changes in the overall configuration can lead to large changes in performance. Third, grouping of elements is key. Only if we know how elements group across the entire visual field, can we determine performance on individual elements, i.e., challenging the classical stereotypical filtering approach, which is at the very heart of most vision models.

Object recognition traditionally proceeds from the analysis of simple to complex features. The Gestaltists proposed a number of basic rules, such as spatial proximity and good continuation, that underlie the grouping of elements into objects. Whereas the Gestalt rules work well for very basic stimuli, they grossly fail for slightly more complex stimuli. For this reason, research on Gestalt principles almost disappeared after the 1930's. After world war II, the discovery of the receptive field advanced vision science by revealing fundamental principles of retinal and cortical processing, which has led to a core scenario that is often, explicitly or implicitly, behind most models in visual neuroscience and the psychology of perception, and provides the basis for most models in computer vision.

The model is characterized by its hierarchical and feedforward organization (Figure 1). Neurons in lower visual areas, with small receptive fields, are sensitive to basic visual features. For example, neurons in V1 respond predominantly to edges and lines. These neurons project to neurons at the next stage of the hierarchy, which code for more complex features. By V4, the neurons are selective for basic shapes, and by IT they respond in a viewpoint-invariant manner to full objects. Decisions making happens in the frontal cortex. This basic scenario has a well-defined set of characteristics. Processing is hierarchical, feedforward, and local on each level, i.e., only neighboring neurons, coding for neighboring parts in the visual field, project to a common higher-level neuron (Figure 1). In addition, processing at one stage is fully determined by processing at the previous stage. Information lost at previous stages is irretrievably lost. Processing follows an atomistic, Lego® building block type of encoding. For example, a hypothetical “square neuron” is created by feedforward projections from “lower” neurons coding for vertical and horizontal lines (Figure 1; Riesenhuber and Poggio, 1999; Hung et al., 2005; Serre et al., 2005, 2007a,b). Finally, there is an isomorphism between objects of the outer world (e.g., a blue line), basic neural circuitry (analyzing the blue line), and the corresponding percept (“blue line”). And this is exactly the beauty of these models: naturalizing the subjectivity of perception by identifying the basic neural circuits of perception.

**Figure 1:**
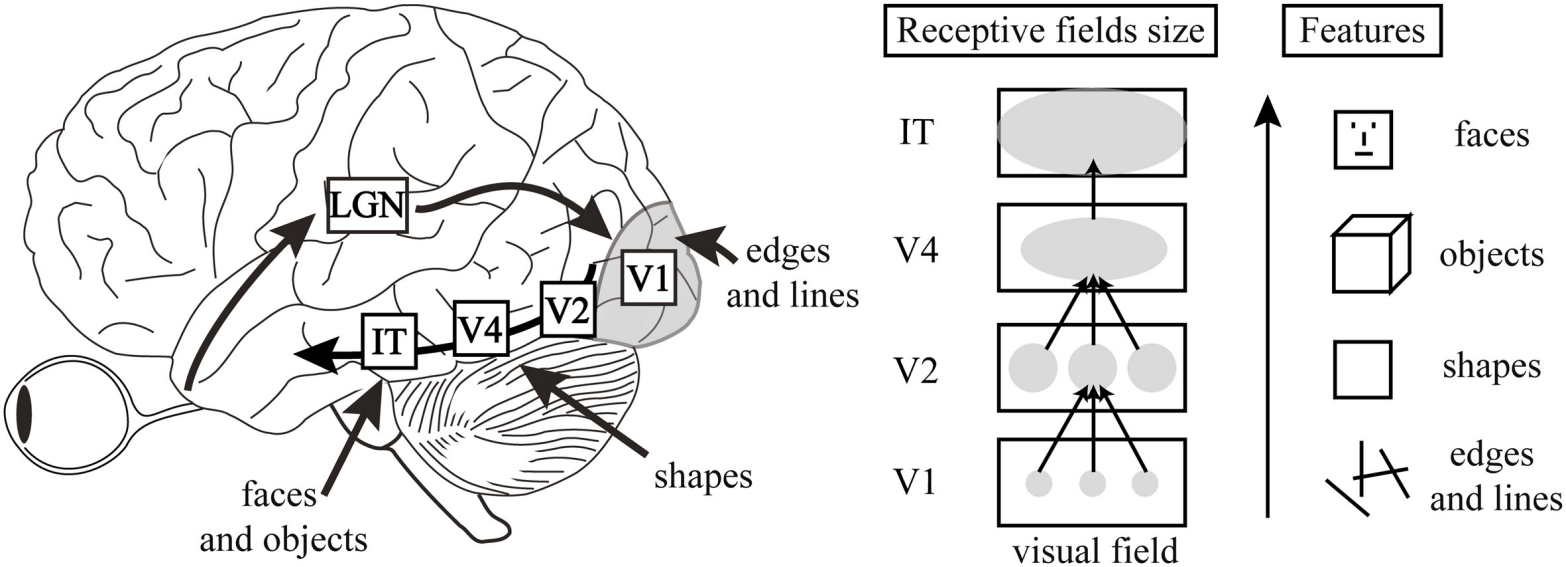
**Left:** A typical hierarchical, feedforward model, where information processing starts at the retina, proceeds to the LGN, then to V1, V2, V4, and IT. Decisions about stimuli are made in the frontal cortex. **Center:** Lower visual areas have smaller receptive fields, while neurons in higher areas have gradually increasing receptive field sizes, integrating information over larger and larger regions of the visual field. **Right:** Lower visual areas, such as V1, code for basic features such as edges and lines. Higher-level neurons pool information over multiple low-level neurons with smaller receptive fields and code for more complex features. There is thus a hierarchy of features. Figure adapted from Manassi et al. (2013).

Evidence for fast, hierarchical feedforward processing comes from experiments showing that humans can detect animals in a scene in less than 150 ms. Calculations based on neural conduction velocity show that there are only one or two spikes per cortical area before a decision is made, arguing strongly against feedback processing (Thorpe et al., 2001).

Computer vision models often follow closely the philosophy of neurobiological feedforward hierarchies. In these, as in neurobiological models, first, basic features are extracted, for example, through V1-style Gabor filtering or Haar wavelets. Often, the downstream hierarchical stages (V2, V4) are collapsed into one processing stage, where a classifier is trained to detect specialized objects such as faces or cars. Similar to IT neurons, these detectors are often scale- and viewpoint-invariant (Biederman, 1987; Ullman et al., 2002; Fink and Perona, 2003; Torralba, 2003; Schneiderman and Kanade, 2004; Viola and Jones, 2004; Felzenszwalb and Huttenlocher, 2005; Fei-Fei et al., 2006; Amit and Trouvé, 2007; Fergus et al., 2007; Heisele et al., 2007; Hoiem et al., 2008; Wu et al., 2010).

Here, we will present experiments from crowding research that challenge classical feedforward hierarchy models. In crowding, target discriminability strongly deteriorates when neighboring elements are presented (Figure 2). Crowding is often seen as a breakdown of object recognition and most models of crowding are very much in the spirit of object recognition models. In pooling models, information from lower-level neurons is pooled by higher-level neurons, to see wholes at the cost of more poorly perceiving the parts. Indeed, observers can clearly *detect* a crowded target, it is only its features and spatial relationships that are jumbled with flanker features (e.g., Pelli et al., 2004). Target-feature perception is lost because target and distracter features are pooled. A prediction made by pooling models is that, because spatial integration is local at each stage, only nearby elements deteriorate target discriminability (Bouma's law). In addition, if more flankers are added within Bouma's window, performance should deteriorate (or at least not improve) because the signal-to-noise ratio decreases. A third prediction is that adding more flankers should deteriorate performance.

**Figure 2:**
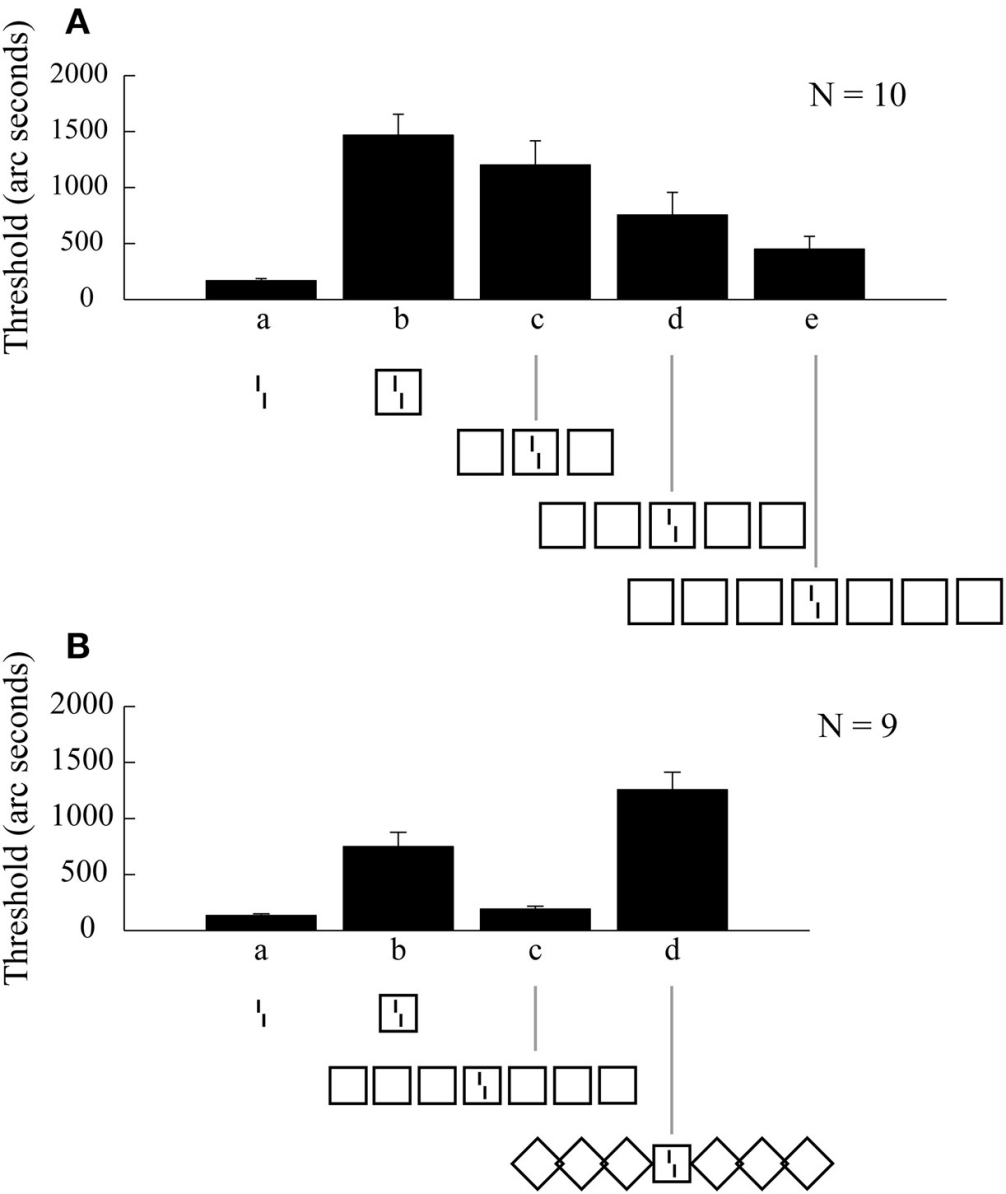
**Vernier offset discrimination as a function of stimulus configuration. (A)**. The reference stimulus is the un-flanked vernier shown in **a**. Enclosing the vernier in a square deteriorates performance. Adding additional squares leads to increasingly better performance. **(B)**. We replicated the results with the squares (b,c). In addition, rotating the flanking squares to form diamonds (d) undoes the effect of grouping and reinstates the crowding effect. From Manassi et al. (2013).

In previous experiments, we presented a vernier stimulus, which consists of two vertical lines, offset slightly to the left or right (Manassi et al., 2012, 2013). Observers indicated the offset direction. Verniers were presented in the periphery, 9 degrees (of visual angle) to the right of fixation. Performance strongly deteriorated when the vernier was surrounded by a square (Figures 2Aa, b). This is a classic crowding effect and is well-explained by traditional crowding models. Next, Manassi et al. (2012, 2013) presented 2 × 3 neighboring squares (Figure 2Ae). According to pooling models, and most object recognition models, more flankers should deteriorate performance. However, the opposite was the case. Crowding almost disappeared. Interestingly, this *un*crowding effect increased with the number of squares that were presented (Figure 2A). Importantly, the fixation dot was only 0.5 degrees apart from the left-most square, i.e., the stimulus configuration extended over large parts of the right visual field. Hence, vernier offset discrimination is influenced by elements far outside the integration region predicted by Bouma's law. Second, and more importantly, vernier offset discrimination is influenced by the overall stimulus configuration. This becomes evident when turning the flanking squares by 90° creating diamonds, resulting in the return of the crowding effect (Figure 2B). Hence, figural aspects determine basic feature processing (Wolford and Chambers, 1983; Livne and Sagi, 2007; Malania et al., 2007; Sayim et al., 2010).

Our results clearly show that simple pooling models cannot explain crowding and the same seems to be true for most basic models of object recognition. Figural processing determines low-level processing as much as low-level processing determines figural processing. It seems that first the squares are computed from their constituting lines. Next square representations interact with each other and the outputs of this processing determine the vernier offset discriminability. This is reminiscent of the famous quote by Wertheimer that “the whole determines the appearance of the parts” (Wertheimer, 1938). In our example, the whole determines even low-level processing. It also agrees with more modern sentiments suggesting that feedback is crucial for normal vision at all levels of the processing hierarchy (Krüger et al., 2013). We propose that it is only when we know how elements group together that we will be able to accurately predict performance on even the simplest tasks, i.e., without understand grouping across the entire visual field, it is impossible to understand human object recognition.

Note here, that we are not claiming that the visual system is not hierarchical. Nor are we claiming that there is no feedforward sweep through the cortex. We are arguing against models that are both feedforward *and* contain a strict feature hierarchy. For example, classic models posit that low-level features (such as verniers) are encoded at an early cortical level and that shapes (such as squares) are encoded at a later cortical level. Square-square interactions are crucial, as we have shown. However, since there are no feedback connections, the classic models cannot explain how square-square interactions change low-level processing of the vernier. One solution is to give up feedforward processing and have *recurrent* interactions between lower and higher levels of processing.

Evidence for recurrent processing comes from timing experiments on the dynamics of grouping in crowding (Manassi and Herzog, 2013; Manassi, 2014). A vernier target was flanked by either two vertical lines, or by two vertical lines that formed the edges of two cuboids. In both cases, the vertical lines were identical and only the surrounding context differed—the lines grouped with the vernier, but when they were part of the cuboids, the lines segmented from the vernier. Vernier offset discrimination thresholds were measured as a function of stimulus presentation time for seven fixed durations ranging from 20 ms to 640 ms. Under brief presentation times (≤120 ms) performance in the two stimulus conditions did not significantly differ. Beyond 160 ms, however, performance with the cuboids was significantly better than with the lines. These results indicate that perceptual grouping evolves with time, even for such basic stimuli as verniers. Current models of vernier offset discrimination show that this task can be achieved in a feedforward way by reading out the responses of orientation-tuned V1 neurons (Wilson, 1986)—a process that takes on the order of 50 ms (Cottaris and De Valois, 1998; Gershon et al., 1998). Sending spikes to additional synapses requires at least 10 ms per spike. Thus, the long time required for vernier discrimination in the cuboid flanker condition to be differentiated from line flanker condition (≥160 ms, i.e., more than double the arrival time of the stimulus at V1) indicates significant additional cortical processing for perceptual grouping. Since 160 ms −50 ms = 110 ms, at least 11 additional synaptic connections could be activated. Recent electrophysiological evidence suggests that the additional time can be accounted for by feedback connections from the lateral occipital cortex to earlier cortical areas, the result of which is the promotion of perceptual grouping (Shpaner et al., 2013).

Our results are not restricted to crowding but occur in many other visual paradigms including overlay masking (Saarela and Herzog, 2008, 2009), backward masking (Herzog, 2007; Hermens and Herzog, 2007; Dombrowe et al., 2009), letter recognition (Saarela et al., 2010), in haptics (Overvliet and Sayim, 2013), and in audition (Oberfeld et al., 2012).

Why does the processing of an element's basic features depend on remote elements? Vision is ill-posed. For example, the light (luminance) that arrives at the retina is a product of the light shining on an object (illuminance) and the material properties of the object (reflectance). Hence, on the photoreceptor level, it is impossible to determine whether or not a banana is yellow and ready to eat. The brain tries to solve this problem by discounting the illuminance, taking contextual information into account. This becomes obvious in the case of computing material properties. Glossy objects, for example, reflect bright spots (specularities) in regions of high curvature. Removal or addition of an object's specularities completely changes the object's perceived material, in spite of the fact that the rest of the object remains the same. To compute the material properties, integrating information across the visual field is crucial: where is the illuminance coming from? What is the shape of the object?

Key then, is that without knowing the whole one cannot know the parts. To the best of our knowledge, very few models adopt this approach of including recurrent processing and effectively integrating information over large parts of the visual field. Not surprisingly, these models are highly effective at modeling human data, not only from crowding, but also from many other areas of cognitive science, hinting at their general ability to explain cortical processing. For example, they effectively explain data pertaining to attention (Tsotsos, 1995; Tsotsos et al., 1995; Cutzu and Tsotsos, 2003; Bruce and Tsotsos, 2005, 2009; Rodriguez-Sanchez et al., 2007), and visual object learning (Bengio et al., 2013; Goodfellow et al., 2013; Salakhutdinov et al., 2013). They also do well at scene-segmentation, where successful models typically use a global approach, such as coarse-to-fine image pyramids (Estrada and Elder, 2006) or normalized cuts over extended graphs (Malik et al., 1999, 2001; Shi and Malik, 2000; Ren and Malik, 2002; Martin et al., 2004), which are leveraged to produce human-like scene segmentations. Here, again, computations are not purely local and feedforward, but rather global and iterative. Grossberg has also produced similar models in terms of their ability to do grouping that extends over a scene (Grossberg and Mignolla, 1985; Dresp and Grossberg, 1997), as has Francis (Francis et al., 1994; Francis and Grossberg, 1995). Future work will show whether these models can explain our particular crowding results.

In summary, there is a wealth of evidence suggesting that cortical processing is not purely hierarchical *and* feed-forward. In order to know how the visual system processes fine-grained information at a particular location it is necessary to integrate information about the surrounding context over the entire visual field. Grouping and segmentation are crucial to understanding vision, and must be understood on a global scale.

## Funding

Grant number 320030_135741.

### Conflict of interest statement

The authors declare that the research was conducted in the absence of any commercial or financial relationships that could be construed as a potential conflict of interest.
